# Exploring the comparative cardiovascular death benefits of sodium–glucose cotransporter 2 inhibitors in type 2 diabetes: a frequentist and Bayesian network meta-analysis-based scoring

**DOI:** 10.3389/fendo.2023.1168755

**Published:** 2023-07-03

**Authors:** Samit Ghosal, Binayak Sinha

**Affiliations:** ^1^ Department of Endocrinology, Nightingale Hospital, Kolkata, India; ^2^ Department of Endocrinology, AMRI Hospital, Kolkata, India

**Keywords:** SGLT-2is, network meta-analysis, CV death, type 2 diabetes, ranking

## Abstract

**Background and aims:**

Cardiovascular death (CV death) is the most objective component of the primary or secondary endpoint in cardiovascular outcome trials (CVOTs) conducted with sodium–glucose cotransporter 2 inhibitors (SGLT-2is). CV death is often incorporated into primary composite outcomes. It is combined with major adverse cardiovascular events (MACEs) in trials with atherosclerotic cardiovascular disease (ASCVD) at baseline and with hospitalization due to heart failure (hHF) in trials with heart failure at baseline. Unlike the primary composites, CV death reduction by itself demonstrated significant variations among the CVOTs with SGLT-2is. Moreover, the impact of the individual agents within the SGLT-2i group on the reduction in CV death has not been explored objectively. This network meta-analysis was undertaken to construct a hierarchy based on indirect pairwise comparisons and rankings among the individual agents within SGLT-2is.

**Methods:**

A Cochrane library-based web search yielded 13 randomized controlled trials for analysis. Stata/BE 17.0 and RStudio 2022.07.1 Build 554 software were used to conduct a frequentist and Bayesian network meta-analysis. The effect size was assessed based on the risk ratio (RR). Ranking of the individual agents was performed with a frequentist approach (P-score and a multidimensional scaling [MDS] rank system) and a Bayesian ranking (surface under the cumulative ranking [SUCRA]).

**Results:**

Regarding the overall data, SGLT-2is reduced the CV death risk by 12% (RR: 0.88, 95% CI 0.80–0.96). All three scoring methods resulted in empagliflozin scoring the highest. There was a 15% RR reduction in CV death (95% CI 0.71–1.02) in the ASCVD and multiple cardiovascular risk factor (MRF) groups and an 11% RR reduction in the HF group, with empagliflozin ranking the highest in the former group and dapagliflozin in the latter.

**Conclusions:**

Empagliflozin ranked the highest compared to the other SGLT-2is in the overall population and the trials including type 2 diabetes (T2D) patients with ASCVD or MRF at baseline, while dapagliflozin ranked the highest in the trials of patients with HF at baseline.

**Systematic review registration:**

https://www.crd.york.ac.uk/prospero/display_record.php?ID=CRD42022381556, identifier CRD42022381556.

## 1 Introduction

The cardiorenal benefits associated with sodium–glucose cotransporter 2 inhibitors (SGLT-2is) are well established (1). Practically all major diabetes, cardiac, and nephrology guidelines recommend SGLT-2is as the first-line drug for the prevention of atherosclerotic cardiovascular disease (ASCVD), heart failure, or progression of diabetic kidney disease in conjunction with good metabolic control (2–4). The initial studies of SGLT-2is were conducted on patients with type 2 diabetes (T2D) and either established atherosclerotic cardiovascular disease (eASCVD) or multiple cardiovascular (CV) risk factors (MRFs) (5–7). The primary endpoint in all these trials was a composite 3-point major adverse cardiovascular event (MACE) comprising cardiovascular death (CV death), non-fatal myocardial infarction (NFMI), and non-fatal stroke (NFS), except for the DECLARE-TIMI 58 trial, where a coprimary endpoint in the form of CV death or hospitalization due to heart failure (hHF) was introduced (6). Due to the differences in the types of populations recruited in these studies, the results were heterogeneous, with MACE and CV death benefits in the EMPAREG Outcomes trial, a MACE benefit in CANVAS, and CV death or hHF coprimary in the DECLARE-TIMI 58 trial. Considering the significant contribution of hHF and renal benefits in these trials, all subsequent trials utilized CV death, hHF, or a renal composite as the primary outcome (8–14). In all these trials, the primary outcome endpoint was achieved. As one of the most objective endpoints, CV death featured in all these studies as part of the primary or key secondary outcome.

The results from all the above-mentioned trials resulted in the American Diabetes Association (ADA)/European Association for the Study of Diabetes (EASD) 2022 guidelines recommending either empagliflozin or canagliflozin as the SGLT-2i of choice for patients with ASCVD or MRF and any of the three (empagliflozin, dapagliflozin, or canagliflozin) for patients with T2D and heart failure or diabetic kidney disease (DKD) (2). Although group specific, these recommendations do not clarify the issues related to the within-group choice of agents. In the absence of head-to-head comparison between the agents, it is incumbent upon the physician to choose among these medications, often arbitrarily.

This network meta-analysis was designed to explore the within-group differences of CV death benefits associated with SGLT-2is in patients with T2D. CV death was chosen over composite endpoints and other stand-alone endpoints because of its objective nature, consistent reporting across the trials, and being the most controversial outcome.

This meta-analysis was designed following the PICO question format (shown below):

P (patient population) = Patients diagnosed with T2D.
I (intervention) = Received drugs belonging to the SGLT-2i group.
C (control group) = Compared to placebo.
O (outcome) = The primary aim was to determine whether there was a hierarchical choice when selecting one of the agents from the intervention arm (I) as far as a reduction in CV death was concerned.

## 2 Materials and methods

This review adhered to the Preferred Reporting Items for Systematic Reviews and Meta-Analyses (PRISMA) statement. Our review protocol was prospectively registered (15).

### 2.1 Search strategy and eligibility criteria

**Figure 1:**
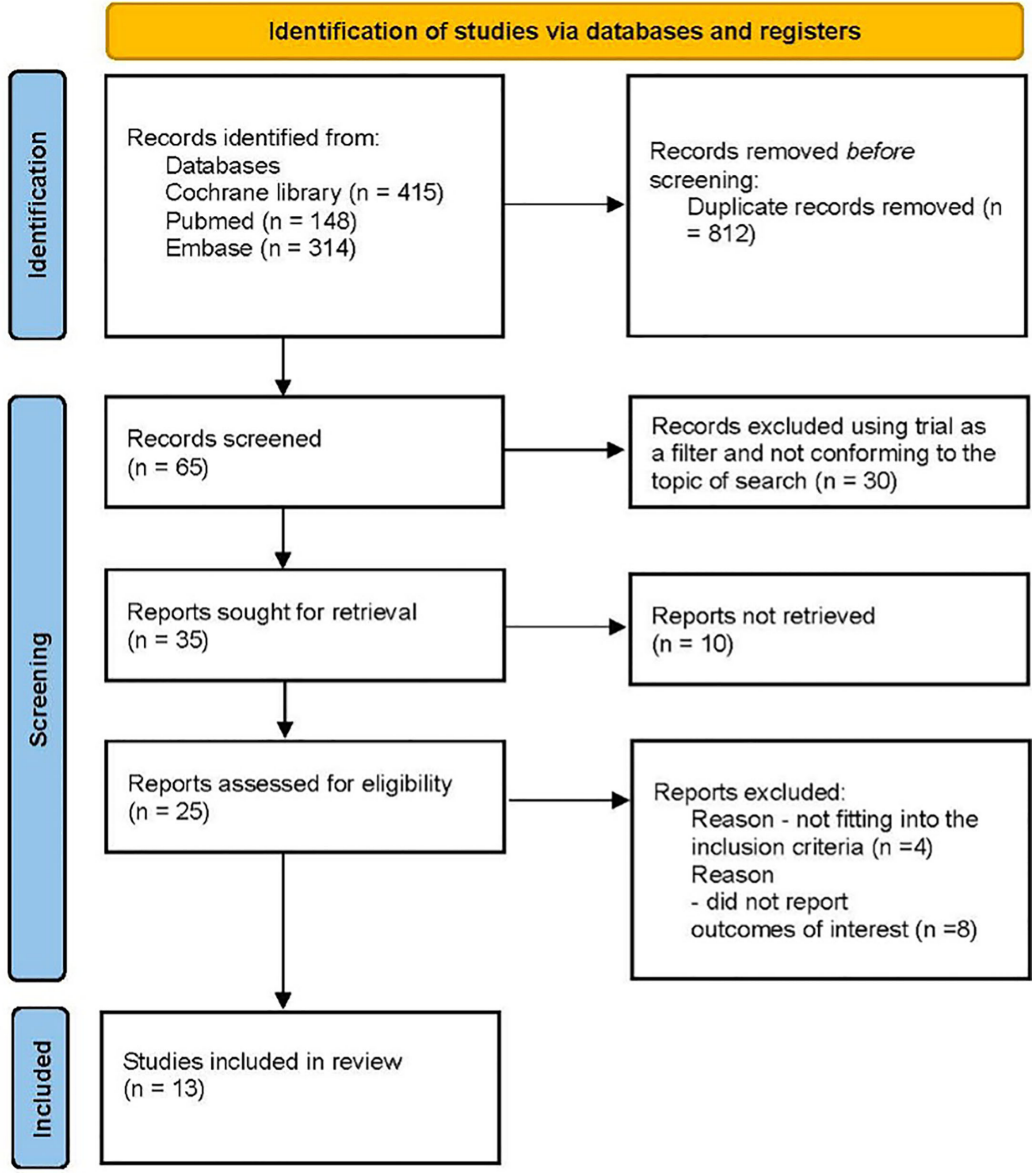
Study selection criteria.

This network meta-analysis was conducted using a predetermined protocol and was reported in accordance with the PRISMA statement. An electronic database search was conducted by the authors (SG and BS) using the Cochrane Library without any limitations on date or language. The search for relevant abstracts presented at major congresses was reviewed manually. The search headings included the following terms [(“type 2 diabetes” {MeSH}, OR “T2D”) OR (“sodium glucose cotransporter 2 inhibitors” {MeSH}, OR “SGLT-2i” OR “empagliflozin” OR “dapagliflozin”, OR “canagliflozin”, OR “ertugliflozin”, OR “sotagliflozin”)] AND [“cardiovascular death”, or “CV death”]. The full search strategy is detailed in **Figure 1**.

### 2.2 Study selection and eligibility criteria

Having performed the preliminary web search, the screened citations were manually sorted by the authors based on the PICO search criteria and the prespecified eligibility criteria. The key eligibility criteria for positive selection included phase 3 randomized controlled trials (RCTs) with the following:

- Patients with T2D.
- Age of the patients ≥18 years.
- Placebo as the comparator arm.
- Studies clearly mentioning the primary outcome of interest that conformed to our intervention requirements: MACE, CV death or hHF, renal composite of CV death as the primary endpoint, and CV death as part of the primary endpoint component or as a key secondary endpoint.

Exclusion criteria:

- Other types of diabetes.
- Acute decompensated metabolic, cardiac, or renal disorders.
- Non-randomized controlled trials.
- Age <18 years.
- Active comparator arm.
- Doses of SGLT-2is used in dose-finding studies. For example, 2.5 mg of dapagliflozin.
- Direct comparison between different doses of SGLT-2is. For example, 100 versus 300 mg of canagliflozin.

### 2.3 Data extraction

Both authors (SG and BS) conducted the database search and literature screening independently and compared the output. After the removal of the duplicate citations and those not conforming to the predetermined inclusion criteria after screening the titles and abstracts, the full-text articles were screened manually. Any disagreement between the authors was settled with mutual agreement.

### 2.4 Risk of bias

The Cochrane Collaboration Risk of Bias 2.0 algorithm was used to assess and report the bias associated with the individual studies (**Supplementary Figure A (2)**). The citations were assessed by all the authors, and any dispute was resolved with consensus. Publication bias was assessed qualitatively using a funnel plot and quantitatively using Peter’s method (**Supplementary Figure A (1)**).

### 2.5 Statistical analysis

The primary objective was to create a hierarchical model to assist the concerned physician in choosing among the different approved SGLT-2is for patients with T2D to reduce their risk of CV death.

Two software programs were used for performing the analysis and preparing the graphical data. The RStudio 2022.07.1 Build 554 software was used to perform the Bayesian network analysis. Stata/BE 17.0 software was used to conduct the pairwise meta-analysis. In addition, multidimensional scaling (MDS) ranking was performed with STATA. With *a priori* power calculation assuming a minimal difference in effect size (risk ratio (RR)) of 10%, from at least 10 studies with 5,000 participants in each arm, an alpha of 0.05, and a moderate degree of heterogeneity, the estimated power of this meta-analysis was 100% (**Supplementary Figure A (3)**).

The planned statistical analysis included the following steps:

- We planned to perform a baseline meta-analysis using the overall (empagliflozin, dapagliflozin, and canagliflozin) database and a subgroup analysis using the individual agents. The primary aim was to identify the quantum of benefit as evident from the effect size, along with an estimation of the precision interval indicating the accuracy of the measurement and the prediction interval indicating the presence of heterogeneity (if any). The degree of overlap of the precision intervals while comparing the individual agents versus placebo was noted.
- The second step would involve conducting a pairwise network meta-analysis using the overall data and then with the individual subgroups (ASCVD or MRF and those with HF).
- The third step included non-reshaped scoring using the frequentist approach (P scores) and reshaped scoring using both a frequentist (MSD rank score) and Bayesian (surface under the cumulative ranking [SUCRA]) approach (**Supplementary Appendix B–D**). This strategy was planned to be applied to the overall population and subsequently to both the subgroups.

The rationale behind including the different scoring systems for assessment was based on the consensus that a P score (without reshaping of data) creates a rank using the effect size as the quantum of benefit, while both the SUCRA and MDS ranking considers the precision interval in addition to the effect size differential. Any discrepancy between the rankings would not allow us to reach a definitive conclusion and hence would be discarded. The top ranking was considered significant if there was concordance between the pairwise comparisons and among all three scoring methods. This strategy would be extremely conservative, and any agent able to overcome this significant burden would be considered for discussion.

## 3 Results

### 3.1 Baseline characteristics of the studies

An electronic database search yielded 13 citations that were included in the network meta-analysis (5–14, 16–18). There were 74,804 patients included in the analysis, with 40,691 in the intervention arm (SGLT-2is) compared to 34,113 in the placebo arm. The mean age of the participants ranged between 62 and 71 years. The primary endpoint was 3-P MACE in four trials (EMPAREG, DECLARE TIMI-58, VERTIS-CV, and CANVAS). DECLARE TIMI-58 had a coprimary endpoint (CV death or hHF) analyzed alongside MACE. CV death or hHF was the primary endpoint in seven trials (EMPEROR-Preserved, EMPEROR-Reduced, DECLARE TIMI-58, DAPA-HF, DELIVER, SOLOIST-WHF, and SCORED). Three trials (EMPA-Kidney, DAPA-CKD, and CREDENCE) had renal composite or CV death as the primary endpoint. CV death was part of the primary component in nine trials (EMPAREG, EMPEROR-Preserved, EMPEROR-Reduced, DECLARE TIMI-58, DAPA-CKD, DAPA-HF). DELIVER, CANVAS, and CREDENCE), while it was part of the secondary outcomes in four trials (EMPA-Kidney, VERTIS-CV, SOLOIST-WHF, and SCORED) (**Table 1**).

**Table 1:** Baseline characteristics of the studies included for analysis.

Study	Year	Intervention	Intervention/placebo (n)	Mean age	Study duration (years)	ASCVD/MRF (%) Intervention arm (entire cohort)	HF (%)	Primary endpoint	CV death status
EMPA KIDNEY (10)	2022	Empagliflozin	1,525/1,515	63.9 ± 13.9	2.0	26.1/73.9	NR	Kidney disease progression or cardiovascular death	Other secondary outcomes
EMPAREG (5)	2015	Empagliflozin	4,645/2,323	63.0 ± 8.6)	3.1	99.5/0.5	9.9	3-P MACE	Primary component
EMPEROR PRESERVED (8)	2021	Empagliflozin	1,466/1,472	70.9 ± 9.0	2.1	36/64	100 (heart failure with preserved ejection fraction [HFpEF])	CV death or hHF	Primary component
EMPEROR REDUCED (9)	2022	Empagliflozin	927/929	67.6 ± 11.6	1.3	52.8/47.2	100 (heart failure with reduced ejection fraction [HFrEF])	CV death or hospitalization due to worsening HF	Primary component
DECLARE TIMI-58 (6)	2019	Dapagliflozin	8,582/8,578	63.9 + 6.8	4.2	40.5/59.5	9.9	3-P MACE and CV death or hHF	Primary component
DAPA-HF (11)	2019	Dapagliflozin	1,075/1,064	66.2 + 11.0	1.5	55.5/36.1 [unknown: 8.4]	100 (HFrEF)	CV death or hospitalization due to worsening HF	Primary component
DAPA-CKD (13)	2020	Dapagliflozin	1,455/1,451	61.8 ± 12.1	2.4	37.8/62.2	10.9	Renal composite or CV death	Primary component
DELIVER (12)		Dapagliflozin	1,578/1,572		2.3	NR	100 (HFpEF)	CV death or hospitalization due to worsening HF	Primary component
CANVAS (7)	2017	Canagliflozin	5,795/4,347	63.2 + 83	3.6	64.8/35.2	13.9	3-P MACE	Primary component
CREDENCE (14)	2019	Canagliflozin	2,202/2,199	62.9 ± 9.2	2.6	50.5/49.5	14.9	Renal composite or CV death	Primary component
VERTIS-CV (16)	2020	Ertugliflozin	5,499/2,747	64.4 ± 8.1	3.5	100	23.4	3-P MACE	Key secondary outcome
SOLOIST-WHF (17)	2121	Sotagliflozin	608/614	69 (median)	0.75	NR [ASCVD was an exclusion criterion]	79.1	CV death or hospitalization due to worsening HF	Secondary endpoint
SCORED (18)	2021	Sotagliflozin	5,292/5,292	69 (Median)	1.3	11.5/88.5	31	CV death or hospitalization due to worsening HF	Major secondary endpoint

### 3.2 Baseline meta-analysis including subgroup analysis

The pooled data from all 13 citations demonstrated a 12% relative risk (RR) reduction compared to the placebo with a 95% precision interval of 0.80–0.96. However, there was significant heterogeneity in the outcome, as evidenced by a prediction interval ranging between 0.69 and 1.12 (**Supplementary Figure A [4: a(i)]**). A sensitivity analysis was performed using the leave-one-study strategy to explore whether a particular study skewed the pooled effect size. In view of the similarity of the effect size, precision interval, and prediction interval of the sensitivity analysis, we went ahead with the subgroup analysis (**Supplementary Material E**).

The p-value for interaction was not significant (p = 0.85) in the agent-type subgroup analysis. The RR reduction was the highest (20%, 95% CI 0.57–1.14) in the empagliflozin arm and the lowest in the canagliflozin arm (7%, 95% CI 0.12–7.35) (**Supplementary Figure A [4: a(ii)]**).

When the inclusion criteria were T2D with established ASCVD or MRF (renal insufficiency included as a CVD risk factor), there was a 15% RR reduction in CV death compared to placebo with a precision interval of 0.71–1.02. The agent-specific subgroup analysis resulted in a maximum 35% RR (95% CI 0.30–1.39) reduction in CV death with empagliflozin and a minimum of 7% with canagliflozin (95% CI 0.12–7.35). The p-value of interaction between the subgroups was significant at p < 0.01 (**Supplementary Figure A [4: b(i) & (ii)]**).

Including trials with HF as the primary inclusion criteria, there was an 11% RR reduction in CV death compared to placebo (95% CI 0.83–0.96). There was significant heterogeneity associated with the outcomes (prediction interval 0.77–1.03). The agent-type subgroup analysis resulted in a maximum 17% RR reduction with dapagliflozin (95% CI 0.58–1.20) and a minimum effect size of 5% with empagliflozin (95% CI 0.65–1.35). The p-value for subgroup interactions was significant (<0.01) (**Supplementary Figure A [4: c(i) & (ii)]**).

### 3.3 The network meta-analysis and scoring

All of the included studies had placebo as the comparative arm, and hence, no loop was formed. The network plot had free nodes (**Supplementary Figure A [5]**).

#### 3.3.1 Overall data

The indirect pairwise comparison demonstrated a consistent trend in favor of empagliflozin (**Supplementary Figure A [6 (a)]**).

**Figure 2:**
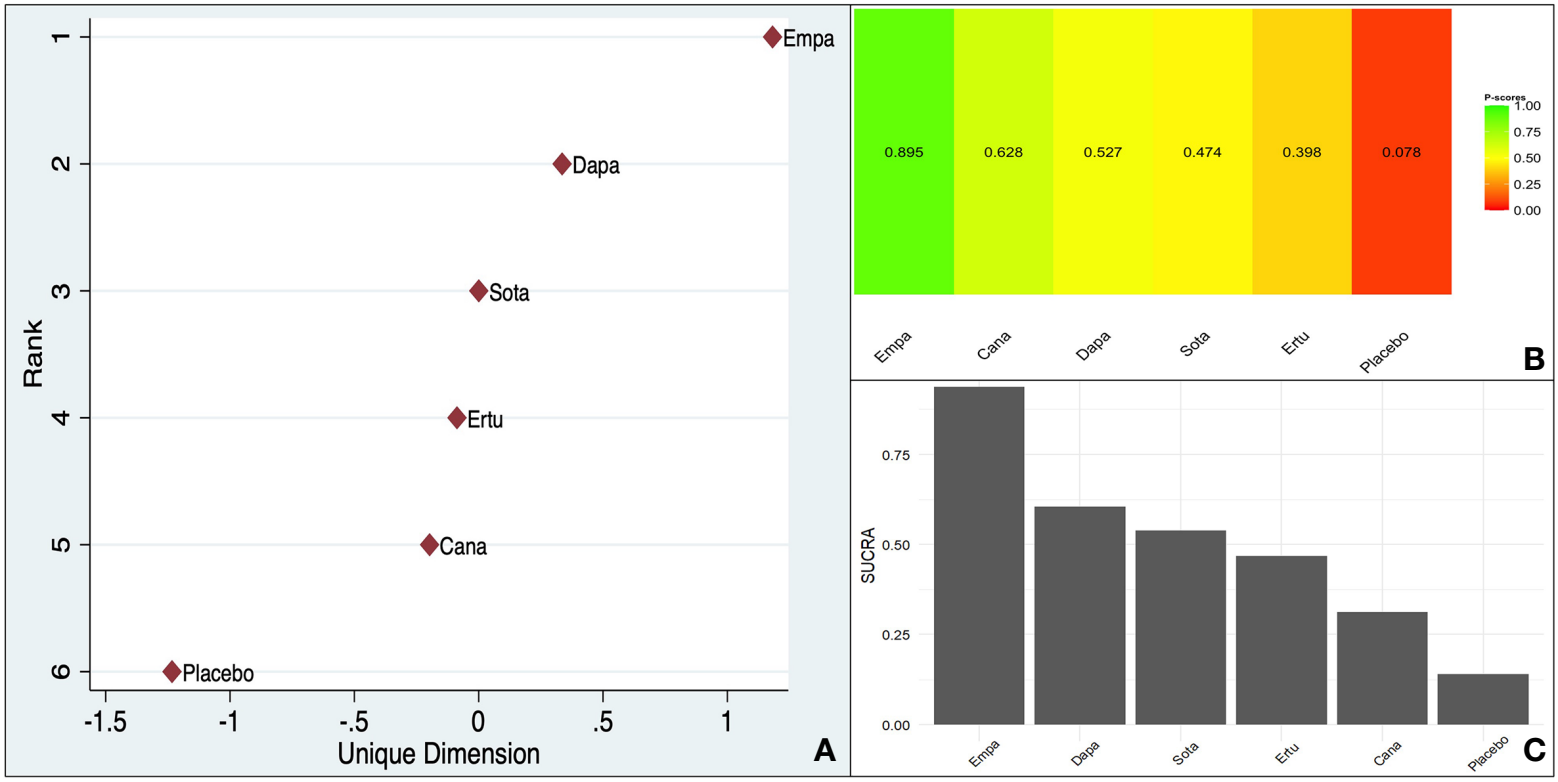
Ranking of different SGLT-2is (Overall data - CV death reduction in the pooled population including ASCVD/MRF & HF): **(A)**. MDS rank score (frequentist), **(B)**. P-score (frequentist), **(C)**. SUCRA (Bayesian).

The frequentist P-score constructed based on the effect size (RR reduction) reflected the pattern encountered in the baseline meta-analysis, with empagliflozin scoring the highest (0.895) among the SGLT-2is as far as a reduction in CV death is concerned. The reshaped frequentist MDS rank scores and the Bayesian SUCRA scores were consistent with the P-score, with empagliflozin scoring the highest (**Figure 2**).

The within-design heterogeneity was significant (Q: 19.84, df: 8, p = 0.01).

#### 3.3.2 ASCVD or MRF

Empagliflozin demonstrated a clear trend favoring it over dapagliflozin (MD 1.45, 95% CI 1.00–2.120) and canagliflozin (MD 1.46, 95% CI 1.01–2.11) (**Supplementary Figure A [6 (b)]**).

**Figure 3:**
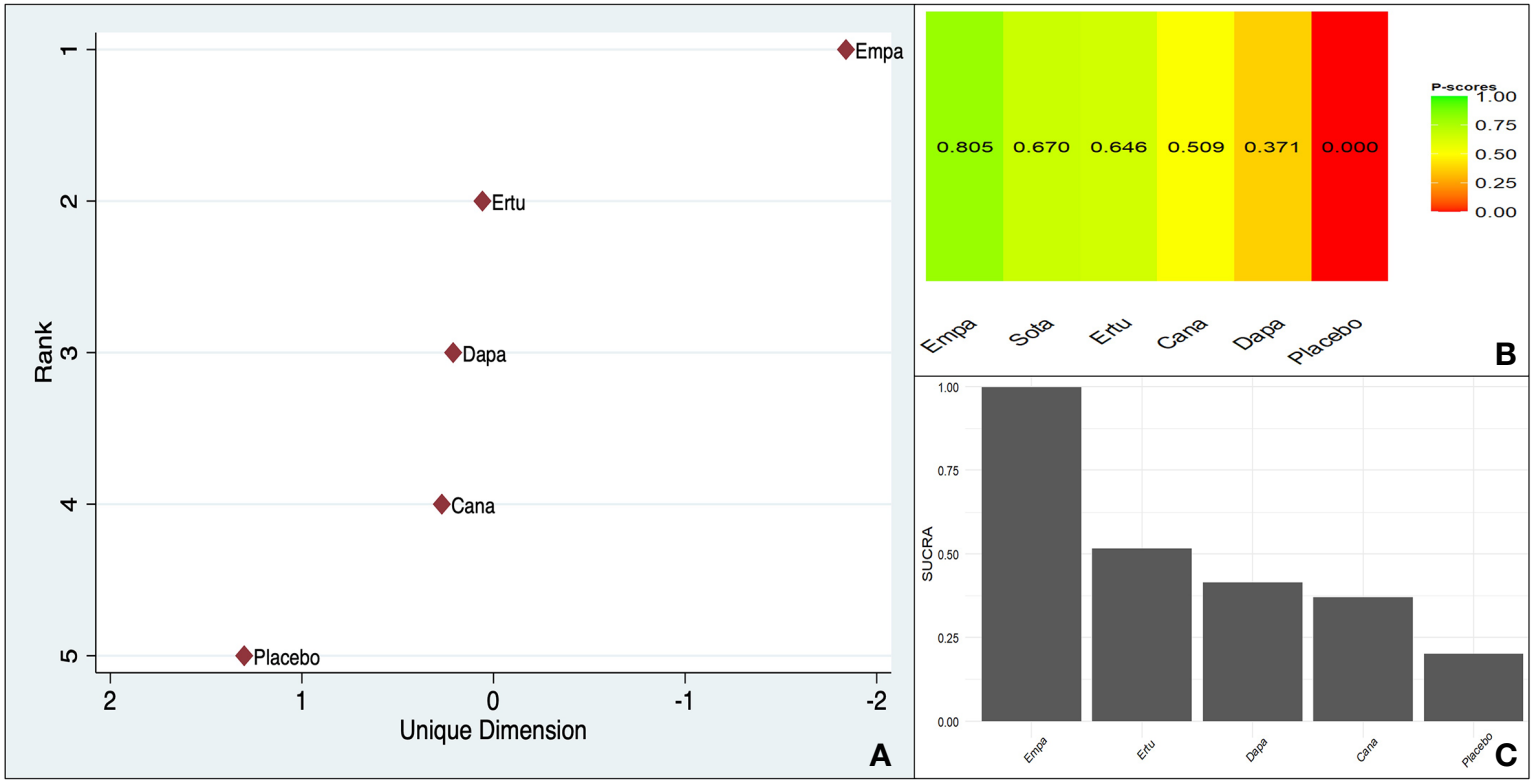
Ranking of CV death benefit of different SGLT-2is taking ASCVD or MRF as the baseline characteristic: **(A)**. MDS rank score (frequentist), **(B)**. P-score (frequentist), **(C)**. SUCRA (Bayesian).

All three scoring systems indicated a clear benefit associated with empagliflozin over all its counterparts. There was a large difference between empagliflozin and the agent ranking second irrespective of the method used (**Figure 3**).

The within-design heterogeneity was not significant (Q: 2.03, df: 3, p = 0.56).

#### 3.3.3 HF

There was no clear trend visible in the comparison versus placebo or the indirect pairwise comparisons (**Supplementary Figure A [6 (c)]**).

**Figure 4:**
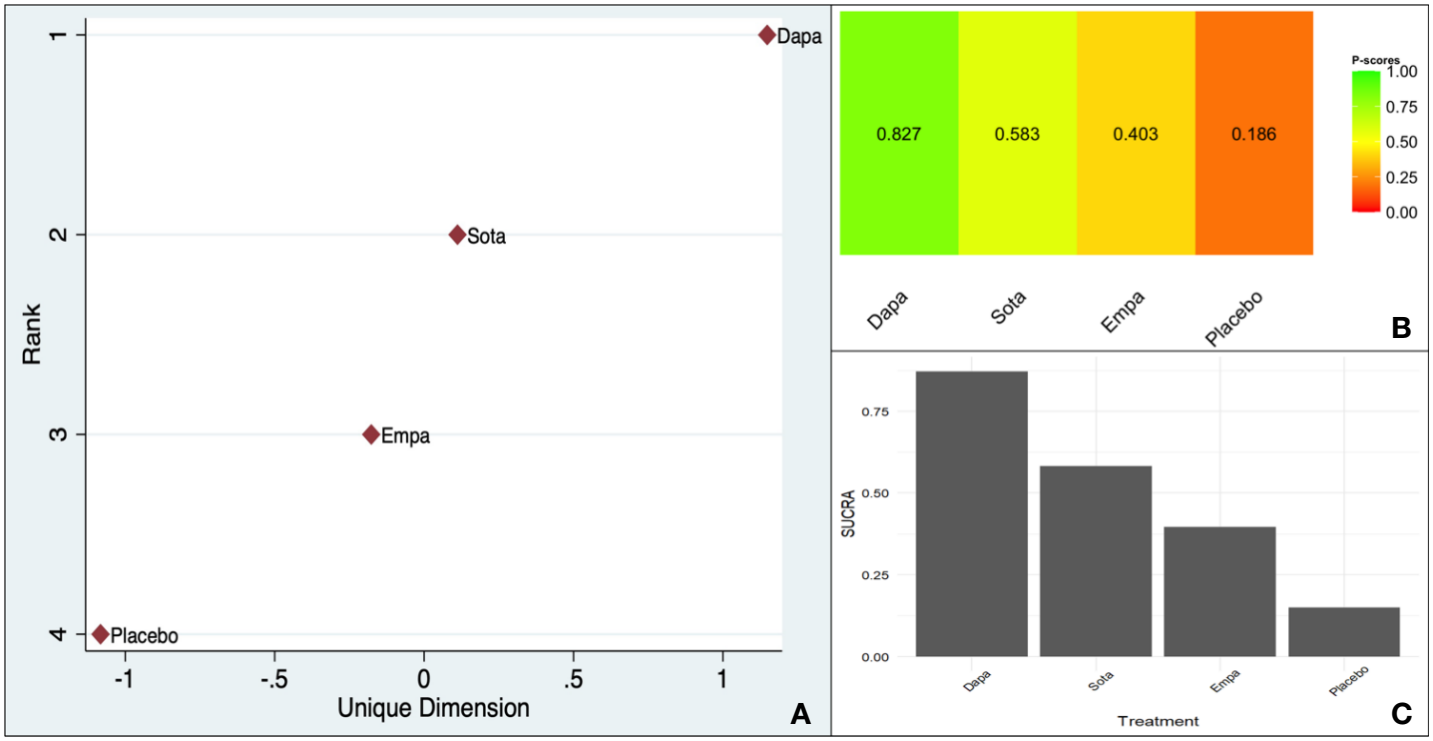
Ranking of CV death benefit of different SGLT-2is taking HF as the baseline characteristic: **(A)**. MDS rank score (frequentist), **(B)**. P-score (frequentist), **(C)**. SUCRA (Bayesian).

However, all three scoring systems were consistent in designating dapagliflozin as the agent securing the highest rank (**Figure 4**).

The within-design heterogeneity was not significant (Q: 0.24, df: 2, p = 0.88).

## 4 Discussion

The cardiorenal benefits of SGLT-2is and GLP1-RAs have revolutionized the management strategy of T2D. The cardiovascular benefits associated with SGLT-2is can be broadly divided into benefits related to the atherosclerotic process or the pumping action of the heart (19). The earlier trials (EMPAREG Outcomes and CANVAS) documented MACE benefits, while all of the recent trials (EMPEROR-Preserved, EMPEROR-Reduced, DAPA-HF, and DELIVER) targeted heart failure as the outcome of prime importance. One of the consistent components of the primary endpoint, MACE, or the heart failure composite was CV death. Being the most objective and clearly defined outcome, CV death reduction has been at the center of academic controversy (20). The only cardiovascular outcome trial (CVOT) to demonstrate CV death benefit as a part of a pre-adjudicated, prespecified primary endpoint component was the EMPAREG Outcomes trial. As a result, empagliflozin was assigned a class IB recommendation as an agent preventing CV death in patients with T2D by the 2019 European Society of Cardiology (ESC) guidelines (3). This outcome was not replicated in any of the subsequent trials recruiting patients with ASCVD or MRF. In contrast, all heart failure trials with SGLT-2is were successful in achieving the primary endpoint of CV death or hHF. However, the stand-alone endpoint of CV death reduction was comparable to a placebo in all the heart failure trials, except for DAPA-HF. Although there was an 18% difference in CV death reduction compared to placebo with dapagliflozin, the outcome was exploratory due to alpha spending.

There are no real controversies, as the primary composite outcome benefits are concerned with the use of SGLT-2is by T2D patients with CV risk (ASCVD as well as HF). However, the CV death benefit effect size is heterogeneous and needs to be explored further.

A recent network meta-analysis (NMA) suggested that empagliflozin had the highest SUCRA-based rank in terms of the reduction in mortality (21). However, that analysis included dose-finding studies that used both standardized doses and doses lower than the clinically approved doses, thus confounding the results. In addition, the distinction between patients with ASCVD/MRF and HF was not explored in this analysis.

This network meta-analysis was undertaken to confirm the findings from a previous meta-analysis (CV death benefits associated with SGLT-2is) with standardized doses and to explore the unchartered area of CV death benefits for T2D patients with ASCVD/MRF or HF.

### 4.1 Findings from our NMA

The baseline pooled meta-analysis indicated a 12% RR reduction (95% CI 0.80–0.96) in CV death compared to placebo, with a non-significant p-value for interaction. However, there was significant heterogeneity, indicating additional factors responsible for the outcome benefit. There was a 15% RR (95% CI 0.71–1.02) and an 11% RR reduction (95% CI 0.83–0.96) in the ASCVD/MRF and HF subgroups, respectively. Empagliflozin scored the highest on the frequentist P score (0.895), in the MDS ranking, and the Bayesian SUCRA scoring of the overall data, with significant within-study design heterogeneity (p = 0.01).

Empagliflozin ranked the highest in the ASCVD and MRF subgroups in all three-ranking systems with non-significant within-study heterogeneity (p = 0.56). This was in accordance with the trend observed from the indirect pairwise meta-analysis.

The picture was less clear in the pairwise network meta-analysis regarding the preferred agent in the HF subgroup. All the three scores were, however, consistent, indicating the superiority of dapagliflozin over all its counterparts with non-significant within-design heterogeneity (p = 0.88).

To summarize, the overall analysis was in favor of SGLT-2is as a group consistent with a recent meta-analysis. However, this conclusion was diluted by the presence of significant heterogeneity (prediction interval 0.69–1.12). Regarding the agent that performed the best in the ranking, our analysis mirrored that of Jiang et al., with empagliflozin emerging as the superior choice. The additional aspect that emerged from our analysis was that dapagliflozin scored the highest in the HF subgroup and empagliflozin scored the highest in the ASCVD and MRF subgroups.

### 4.2 Limitations and strengths

One of the primary limitations of this analysis is the lack of access to individual patient data. The entire analysis was conducted based on published pooled analysis. The design of the indirect pairwise comparison between agents invariably leads to inflation of the confidence interval, which could have led to an underestimation of the pooled effect size. Although the scoring system seems to provide a sense of hierarchy, it is by no means a substitute for a well-conducted head-to-head comparative study. The significant heterogeneity associated with both the overall analysis and the subgroups makes it extremely difficult to identify the use of SGLT-2is as the sole reason for the CV death benefits. The frequent coexistence of ASCVD and HF makes it impossible to choose a single agent in view of empagliflozin being favored for ASCVD and MRF and dapagliflozin for HF. The heterogeneity in the overall data could be related to clinical and biochemical parameters in addition to the differences between the agents used. This network meta-analysis explored the differences between different SGLT-2is as a covariate explaining the heterogeneity of CV death outcomes. However, there is a possibility that other clinical and biochemical parameters could also explain this heterogeneity. To avoid the issue of multiplicity and its associated correction, a single parameter was used as the covariate.

The main strength of this analysis is the very large amount of pooled data included in the analysis. In addition, the inclusion of RCTs as well as a large preanalytical power was an additional strength. Despite significant heterogeneity of the outcome benefit, we cannot deny the role of SGLT-2is in CV death reduction. The additional contributive factors need to be explored. In the absence of planned studies evaluating the comparative effectiveness of SGLT-2is for CV death reduction, a network meta-analysis and scoring seems to be the best available option.

The difference between empagliflozin and dapagliflozin cannot be explained by molecular structure or differences between their pharmacodynamics and pharmacokinetics. A head-to-head 52-week prospective trial found greater glycated hemoglobin (HbA1C) lowering with empagliflozin compared to dapagliflozin but did not find any difference as far as other cardio-metabolic parameters were concerned (22). The only plausible explanation could be related to trial design. With the EMPAREG Outcomes trial recruiting T2D patients with eASCVD in contrast to a mixed population of eASCVD and MRF, the CV death benefits became skewed toward empagliflozin. We could find a very similar trend in the VERTIS-CV trial, where despite not meeting the primary endpoint (MACE), the exploratory endpoint of CV death had a positive trend compared to DECLARE TIMI-58 and CANVAS program.

In the HF trials, CV death as a stand-alone endpoint was exploratory in both the empagliflozin and dapagliflozin trials. The scoring favoring dapagliflozin was a direct result of the positive trend from the DAPA-HF trial. Once again, the differences can only be explained based on the baseline trial designs. Homogeneity of the baseline characteristics across these trials could have resulted in a different inference.

## 5 Conclusion

SGLT-2is is associated with an impressive reduction in CV death in patients with T2D compared to standard of care. Empagliflozin ranked the highest in T2D patients with ASCVD or MRF, and dapagliflozin ranked the highest in T2D patients with HF.

## Data availability statement

The original contributions presented in the study are included in the article/**Supplementary Material**. Further inquiries can be directed to the corresponding author.

## Author contributions

SG and BS conceptualized the study. The meta-analysis was conducted by SG. BS cross validated the data and prepared the manuscript. SG and BS conducted an expanded literature review to contextualize the results from the meta-analysis. All authors contributed to the article and approved the submitted version.
