# Supplementary material for: Exploring the comparative cardiovascular death benefits of sodium–glucose cotransporter 2 inhibitors in type 2 diabetes: a frequentist and Bayesian network meta-analysis-based scoring
Source: Front Endocrinol (Lausanne). 2023 Jul 3;14:1168755. doi: 10.3389/fendo.2023.1168755 (PMC10353048; doi:10.3389/fendo.2023.1168755)
Supplement: Supplementary file 1 [file DataSheet_1.docx]

Supplementary materials

Index

[A]. Supplementary figures

[B]. Appendix 1: STATA code

[C]. Appendix 2: R code

[D]. Bayesian inference: supporting figures

[E]. Leave one out sensitivity analysis

[A]. Supplementary figures:

1. Funnel plot to assess publication bias


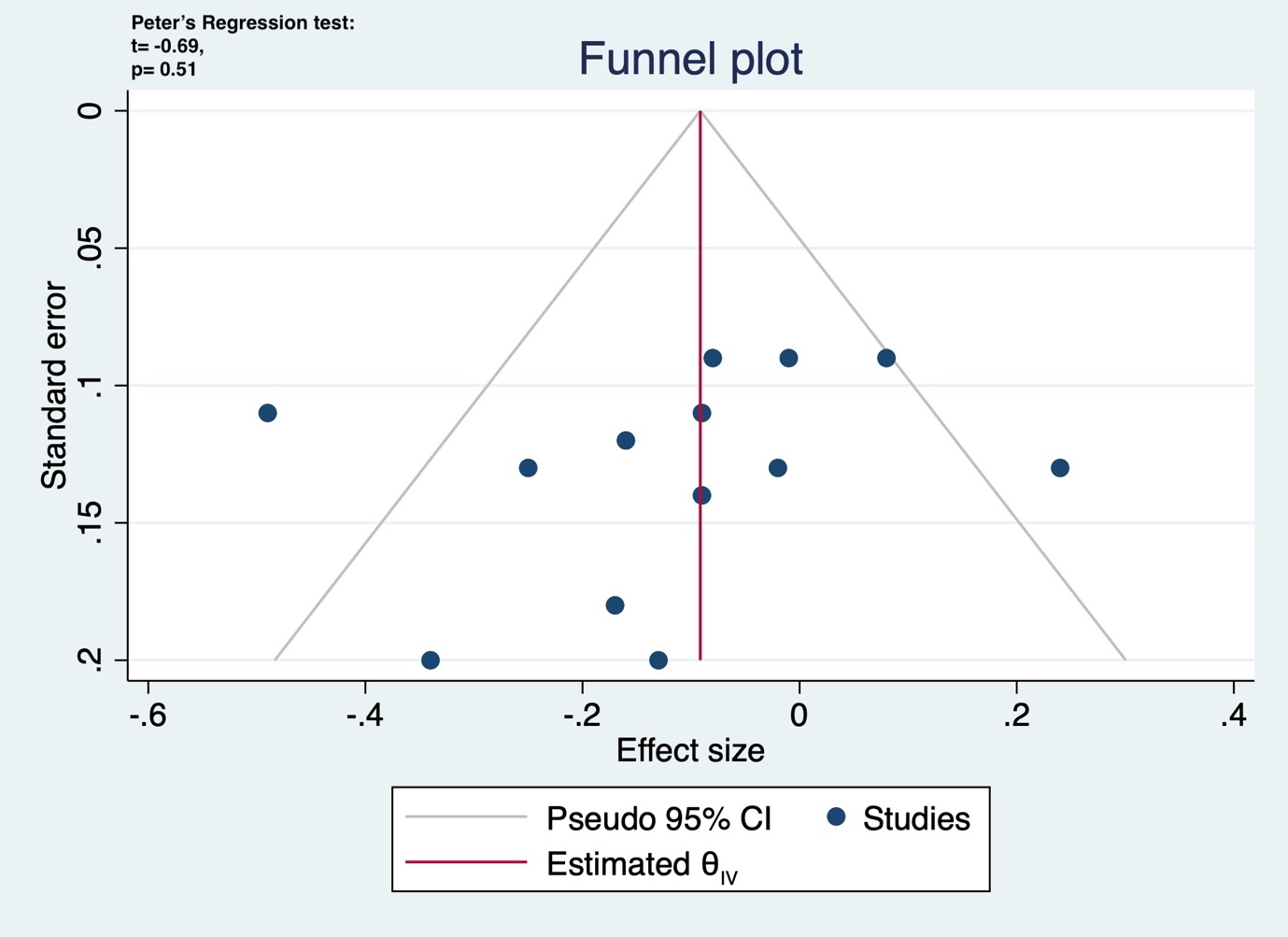


2. Cochrane risk of bias algorithm


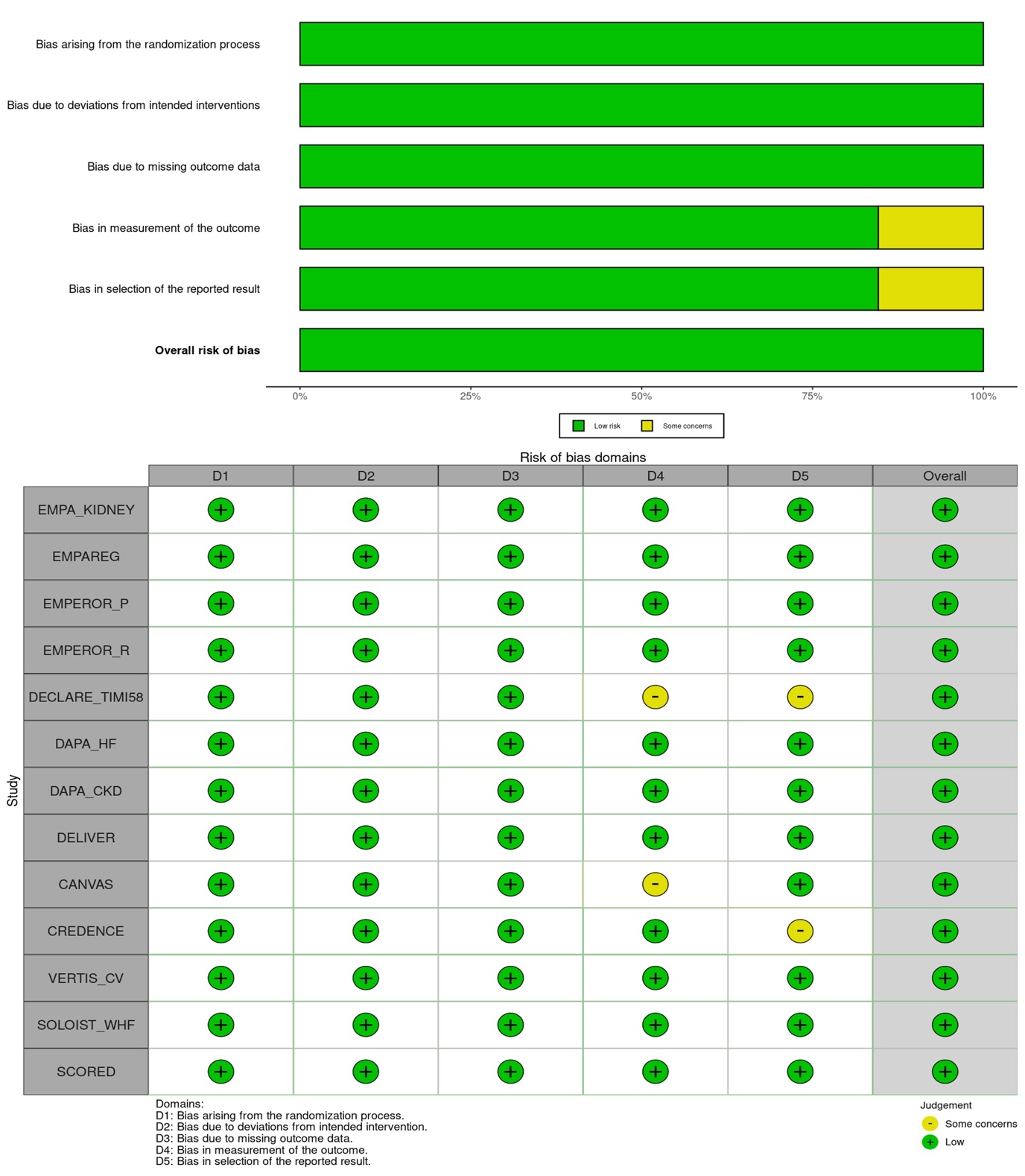


3. Priori power calculation


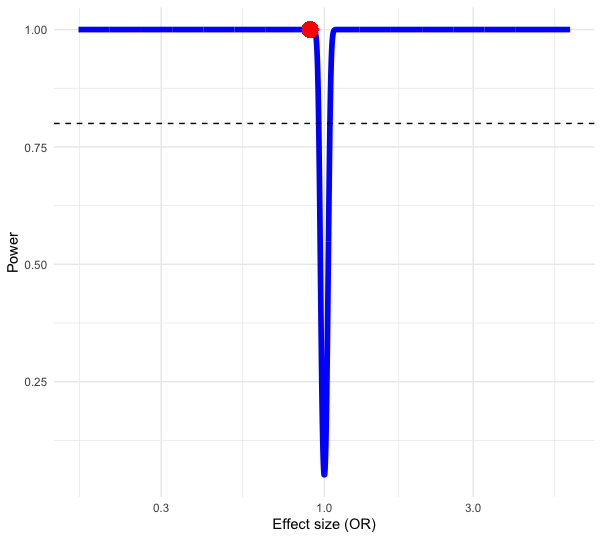


4. Baseline meta-analysis with subgroups

(a). Overall

(i). Overall


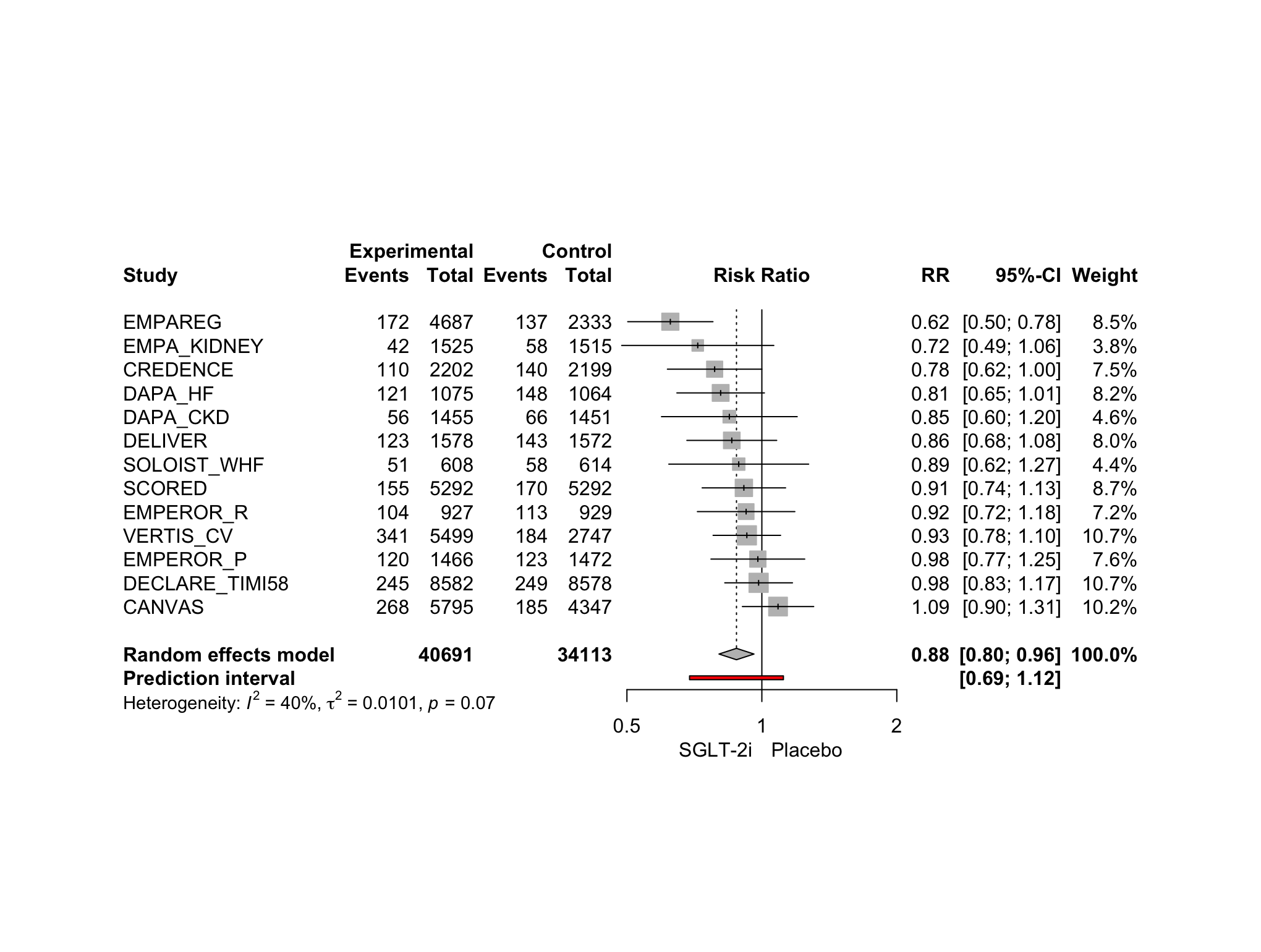


(ii). Subgroup analysis: by agents


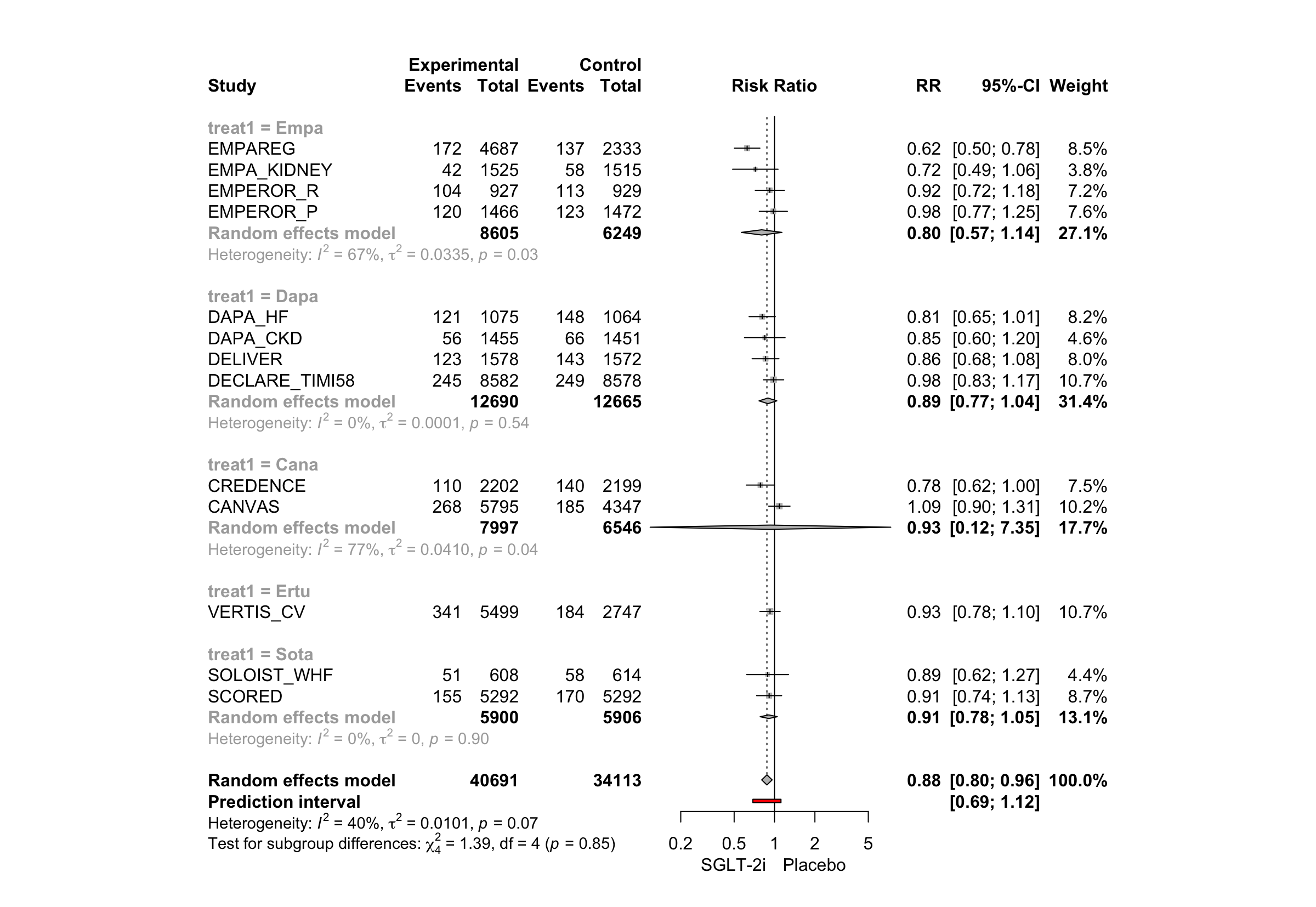


(b). By ASCVD or MRF

(i). Overall:


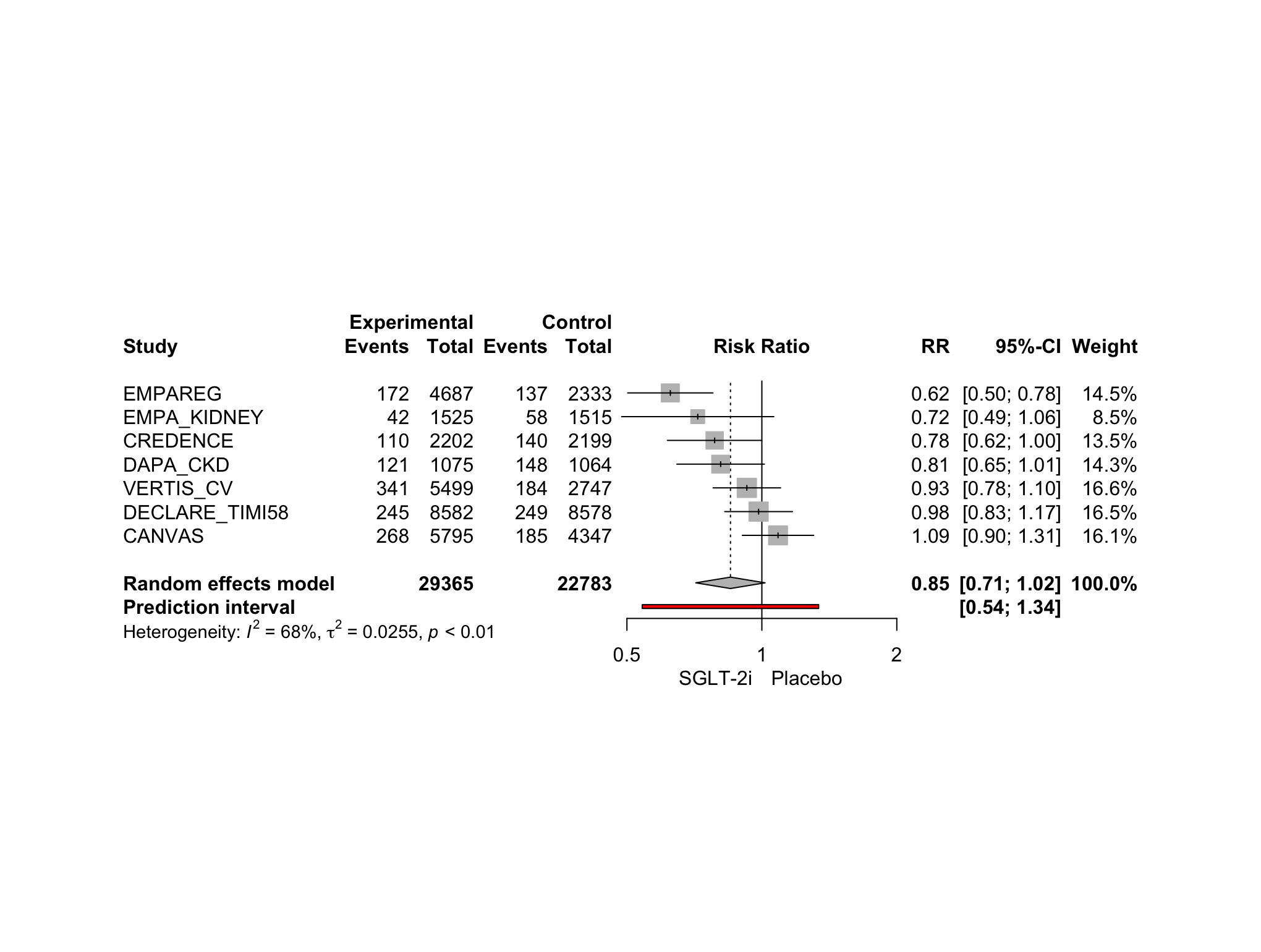


(ii). Subgroup analysis: by agents


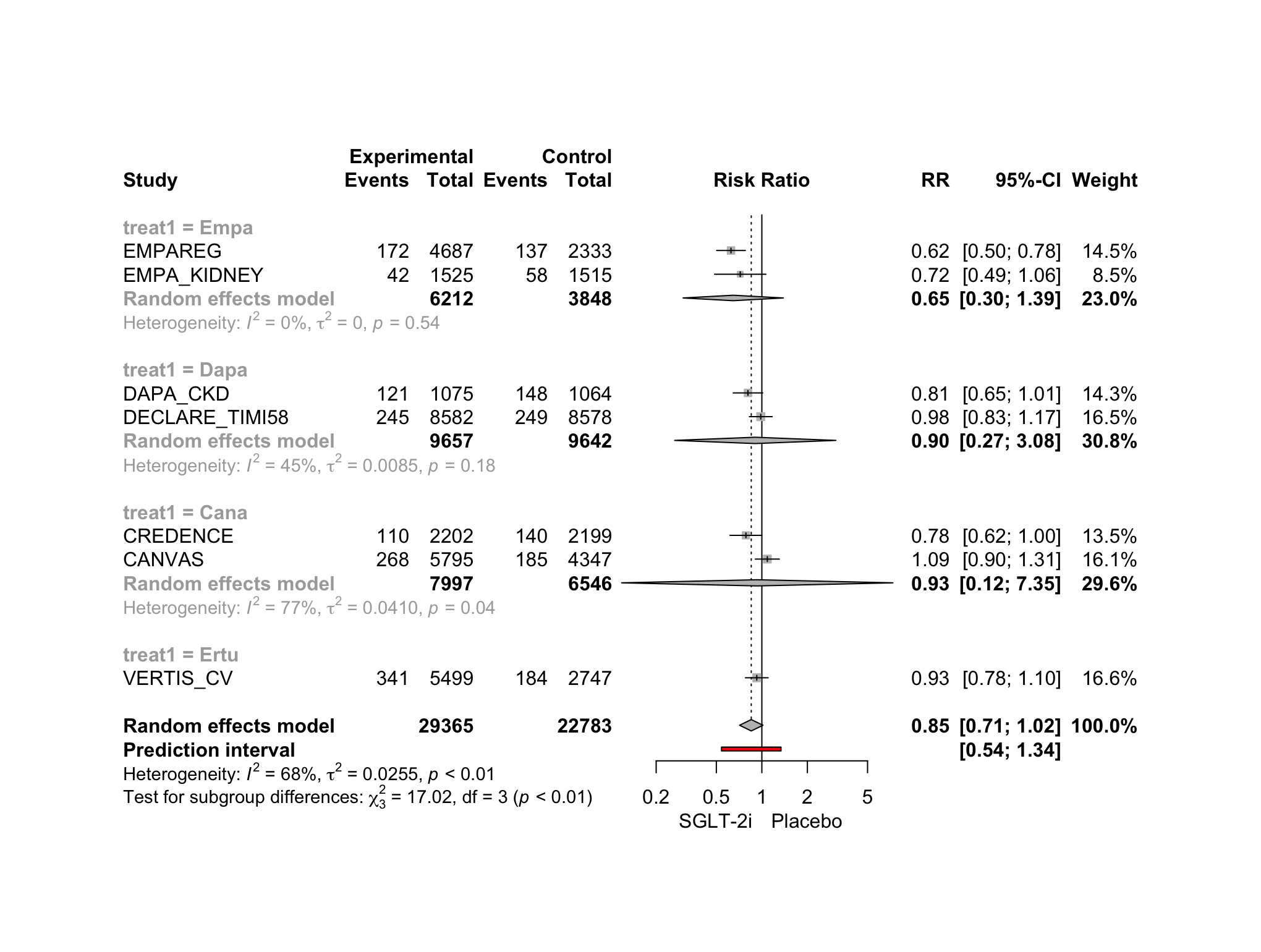


(c). By HF

(i). Overall:


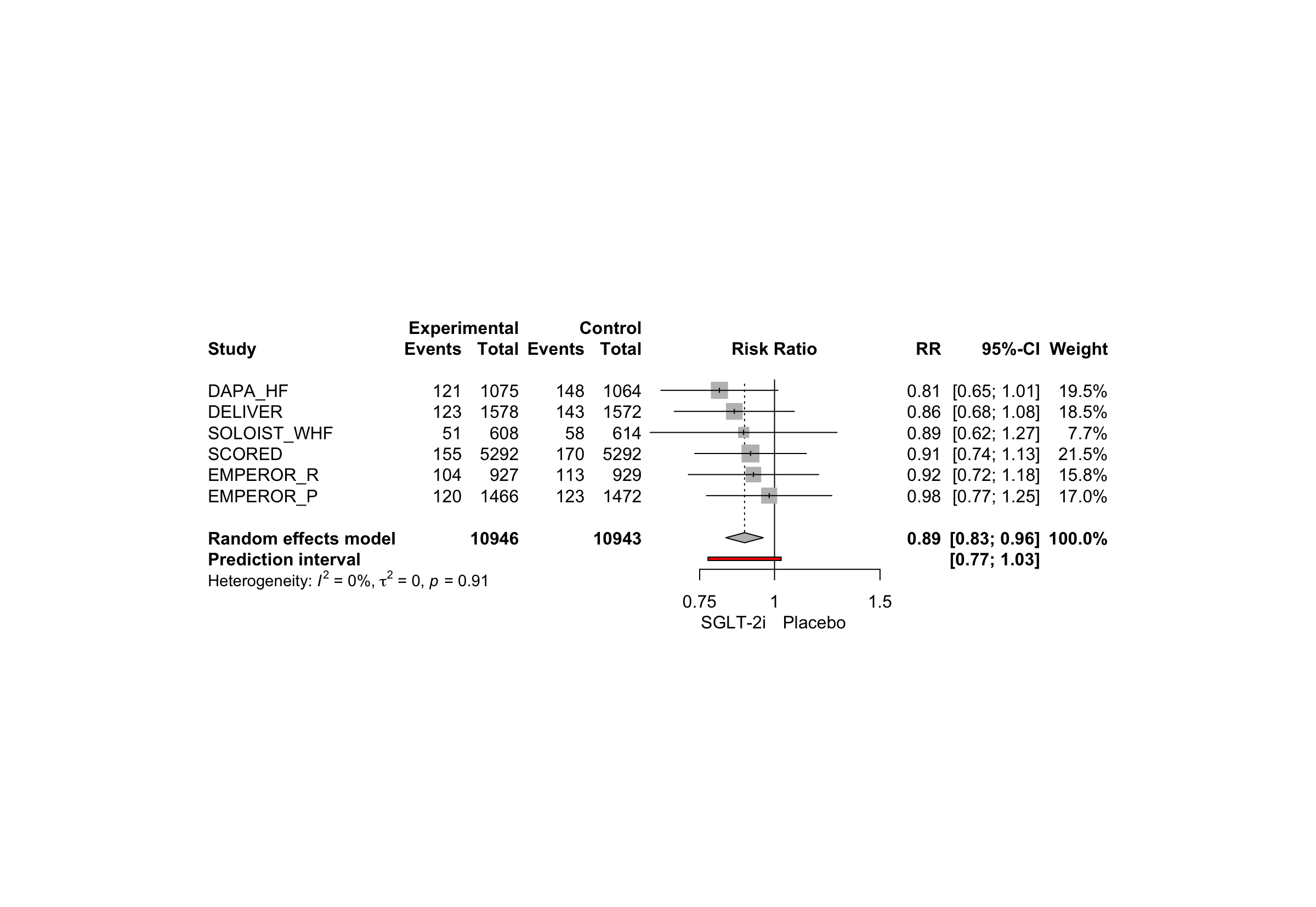


(ii). Subgroup analysis: by agents


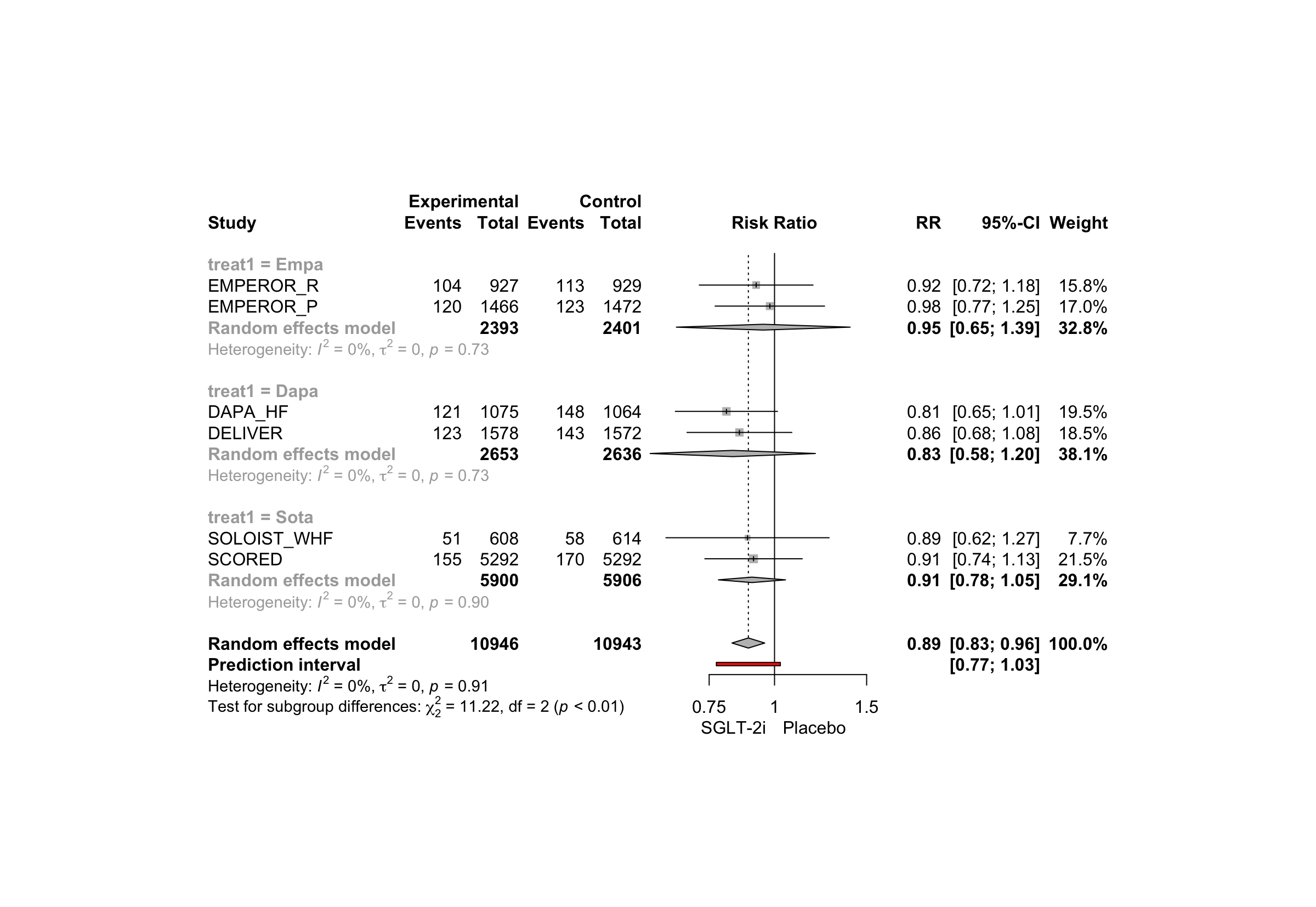


5. Network plot (Overall data):


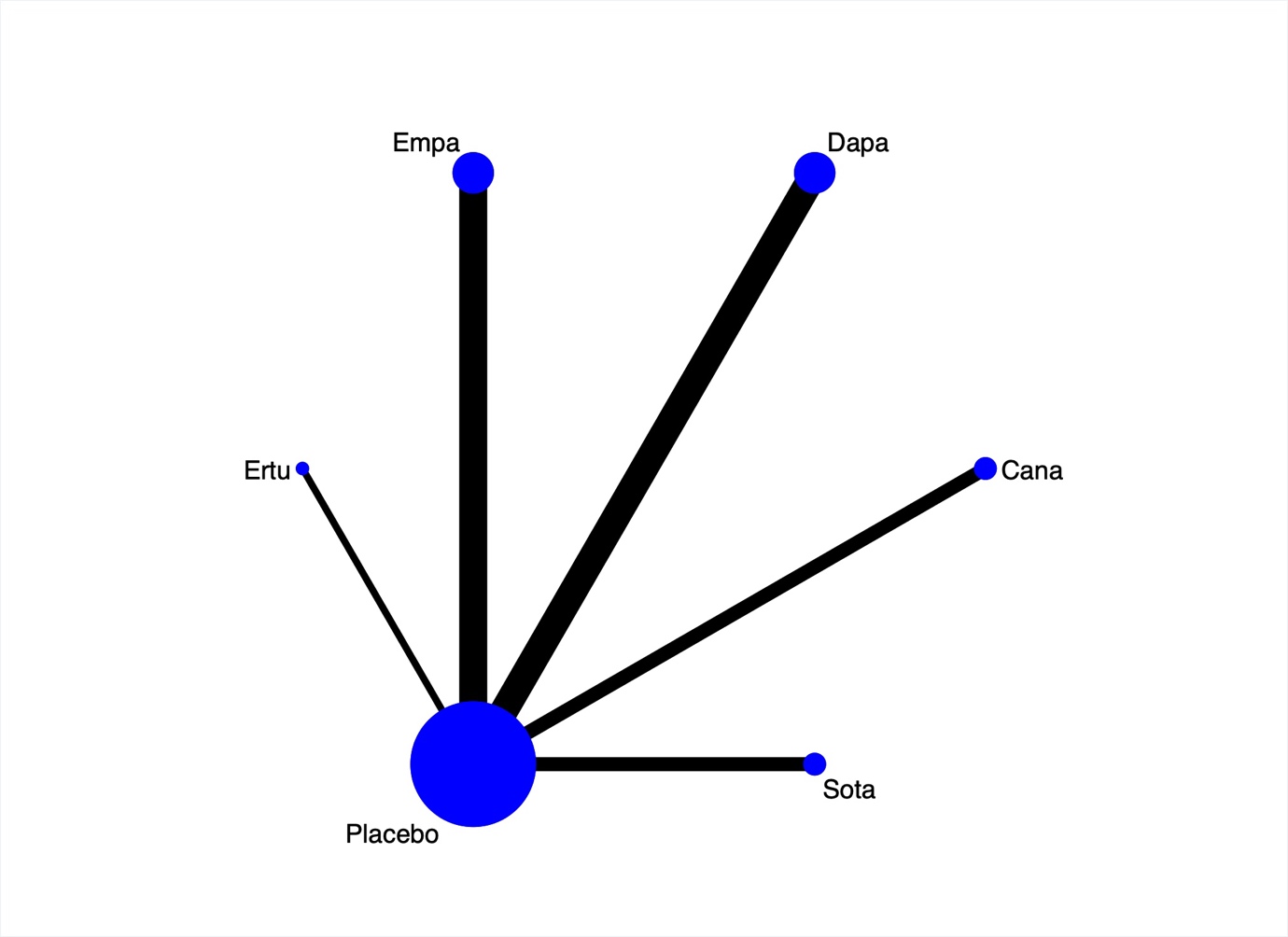


6. Pairwise comparison: Network meta-analysis

(a). Overall:


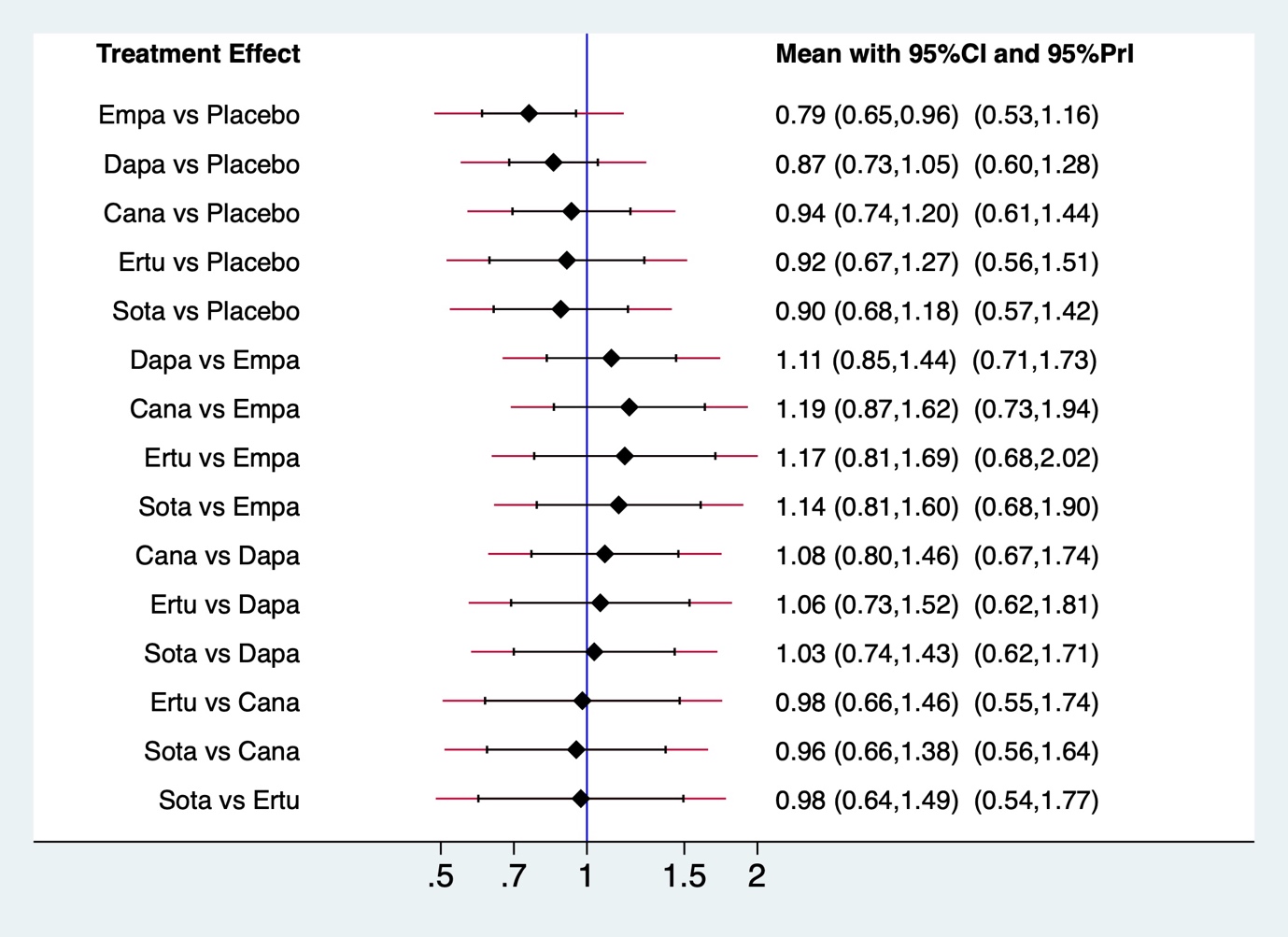


(b). By ASCVD or MRF:


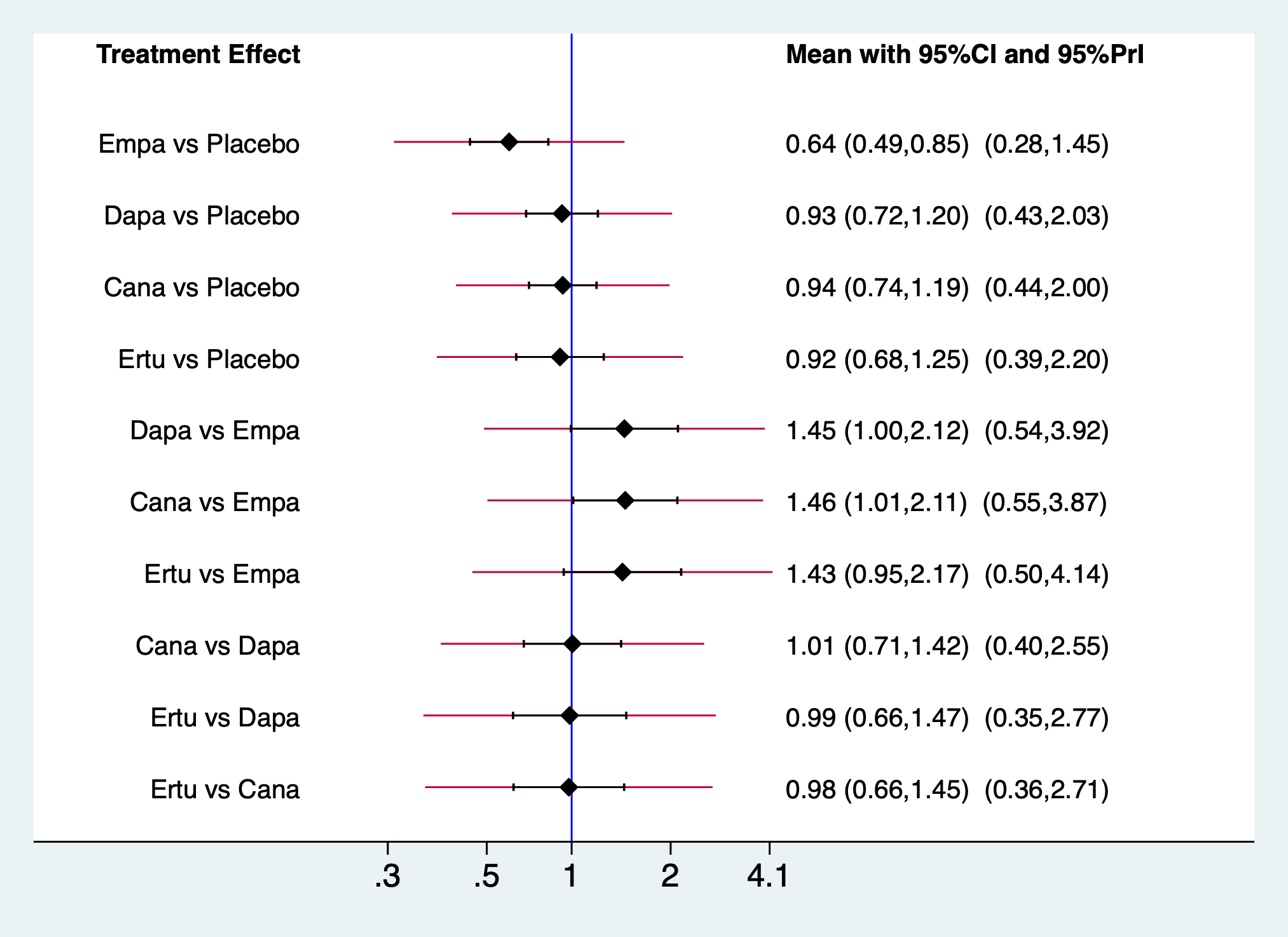


(c). By HF:


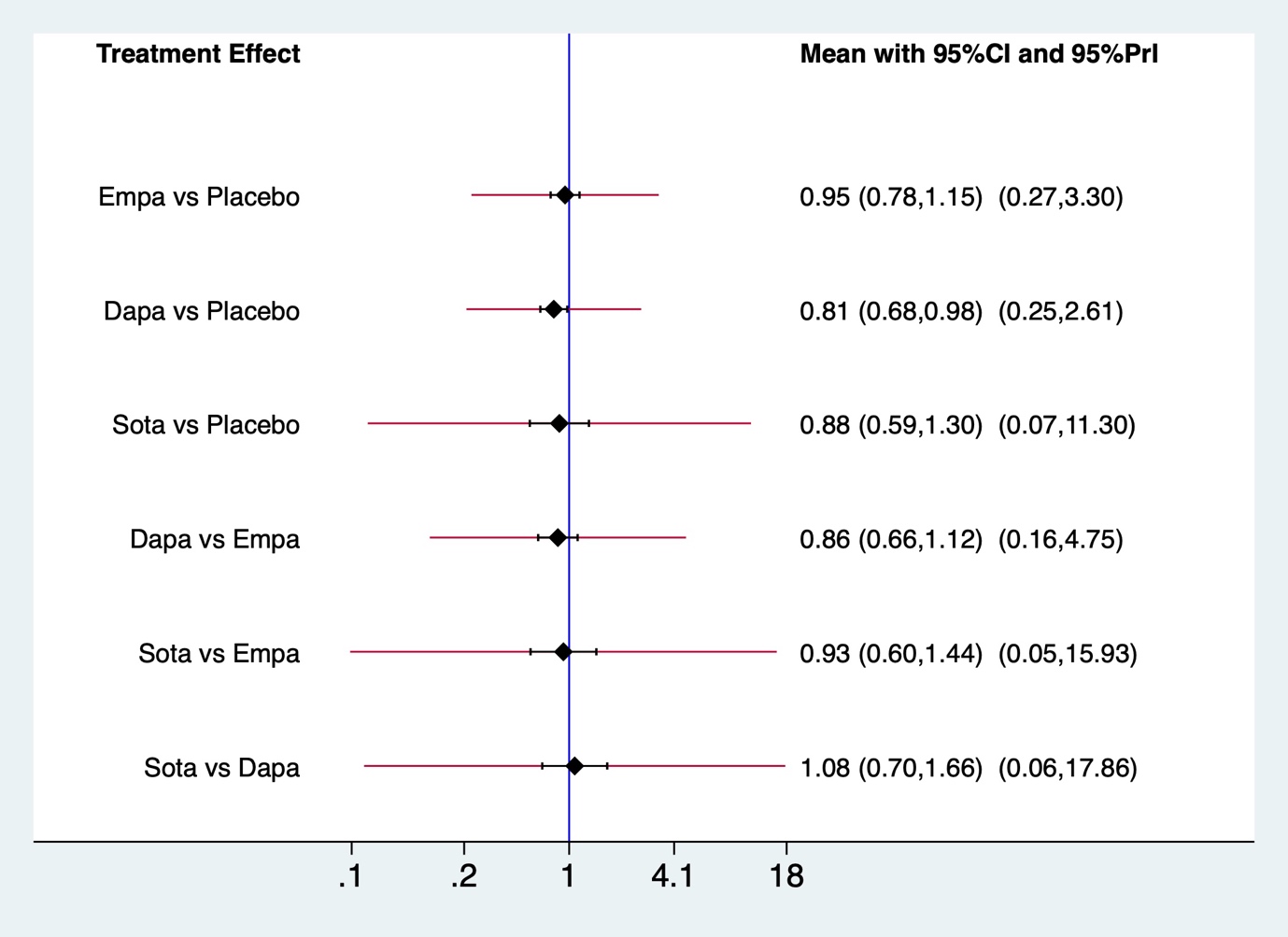


[B]. Appendix 1: STATA code

. drop _all

. import excel "/Users/samit/Desktop/R analysis/CV_Death/By HF/Book2.xlsx",

> sheet("Sheet1") firstrow

(4 vars, 10 obs)

. save "/Users/samit/Desktop/R analysis/CV_Death/By HF/HF.dta"

file /Users/samit/Desktop/R analysis/CV_Death/By HF/HF.dta saved

. use "/Users/samit/Desktop/R analysis/CV_Death/By HF/HF.dta"

. list in 1/5, clean noobs

. network setup r n, studyvar(id) trtvar(t) numcodes ref(1)

. network meta c

. intervalplot, eform pred null(1) labels(Placebo Empa Dapa Sota)

. graph save "Graph" "/Users/samit/Desktop/R analysis/CV_Death/By HF/Graph.g

. netleague, labels(Placebo Empa Dapa Sota)

. intervalplot, labels(Placebo Empa Dapa Sota) noplot notab keep

. split _Comparison, par(" vs ") gen(t)

. list _Comparison _Effect_Size _Standard_Error t1 t2 in 1/5, noobs clean

. mdsrank _Effect_Size _Standard_Error t1 t2 if _Effect_Size!=., best(max)

. graph save "Graph" "/Users/samit/Desktop/R analysis/CV_Death/By HF/Graph3.

> gph"

file /Users/samit/Desktop/R analysis/CV_Death/By HF/Graph3.gph saved

[C]. Appendix 2: R code

1. For Bayesian network analysis with SUCRA

library(gemtc)

install.packages("rjags")

library(rjags)

library(dmetar)

data_b_bin = HF

as.data.frame(HF)

library(tibble)

colnames(data_b_bin) <- c("study", "responders", "sampleSize", "treatment")

network_b_bin <- mtc.network(data.ab = data_b_bin)

plot(network_b_bin)

summary(network_b_bin)

plot(network_b_bin,

use.description = TRUE)

library(igraph)

set.seed(12345) # set seed for reproducibility

plot(network_b_bin, use.description = TRUE, # Use full treatment names

vertex.color = "white", # node color

vertex.label.color = "gray10", # treatment label color

vertex.shape = "sphere", # shape of the node

vertex.label.family = "Helvetica", # label font

vertex.size = 20, # size of the node

vertex.label.dist = 2, # distance label-node center

vertex.label.cex = 1.5, # node label size

edge.curved = 0.2)

model_b_bin_fe <- mtc.model(network_b_bin,

linearModel= "fixed", n.chain=4)

mcmc1 <- mtc.run(model_b_bin_fe, n.adapt = 50,

n.iter = 1000, thin = 10)

mcmc2 <- mtc.run(model_b_bin_fe, n.adapt = 5000, n.iter = 1e5,

thin = 10)

plot(mcmc1)

plot(mcmc2)

gelman.plot(mcmc1)

gelman.plot(mcmc2)

gelman.diag(mcmc1)$mpsrf

gelman.diag(mcmc2)$mpsrf

rank <- rank.probability(mcmc2, preferredDirection = -1)

plot(rank, beside=TRUE, cex.names=0.5)

forest(relative.effect(mcmc2, t1 = "2"))

library(dmetar)

rank.probability <- rank.probability(mcmc2)

sucra <- dmetar::sucra(rank.probability, lower.is.better = TRUE)

plot(sucra)

2. For meta-analysis with prediction interval and P score:

(a). Meta-analysis and subgroup analysis codes:

library(meta)

data = CVD

m.bin <- metabin(event.e = Ee,

n.e = Ne,

event.c = Ec,

n.c = Nc,

studlab = name,

data = data,

sm = "RR",

method = "MH",

MH.exact = TRUE,

comb.fixed = FALSE,

comb.random = TRUE,

method.tau = "REML",

hakn = TRUE,

title = "CV death reduction")

m.bin

forest.meta(m.bin,

sortvar = TE,

prediction = TRUE,

leftlabs = c("study", "g", "SE"),

label.right = "Placebo",

label.left = "SGLT-2i")

sg <- update.meta(m.bin,

byvar = treat1)

forest.meta(sg,

sortvar = TE,

prediction = TRUE,

leftlabs = c("study", "g", "SE"),

label.right = "Placebo",

label.left = "SGLT-2i")

(b). Frequentist network meta-analysis with P score:

library(dmetar)

data = CVD

as.matrix(table(data$study))

m.netmeta <- netmeta(TE = TE,

seTE = seTE,

treat1 = treat1,

treat2 = treat2,

studlab = name,

data = CVD,

sm = "RR",

fixed = TRUE,

random = FALSE,

reference.group = "Placebo",

details.chkmultiarm = TRUE,

sep.trts = " vs ")

summary(m.netmeta)

pscore = netrank(m.netmeta, small.values = "good")

plot(pscore)

[D]. Bayesian inference: supporting figures

1. mcmc1 plot:


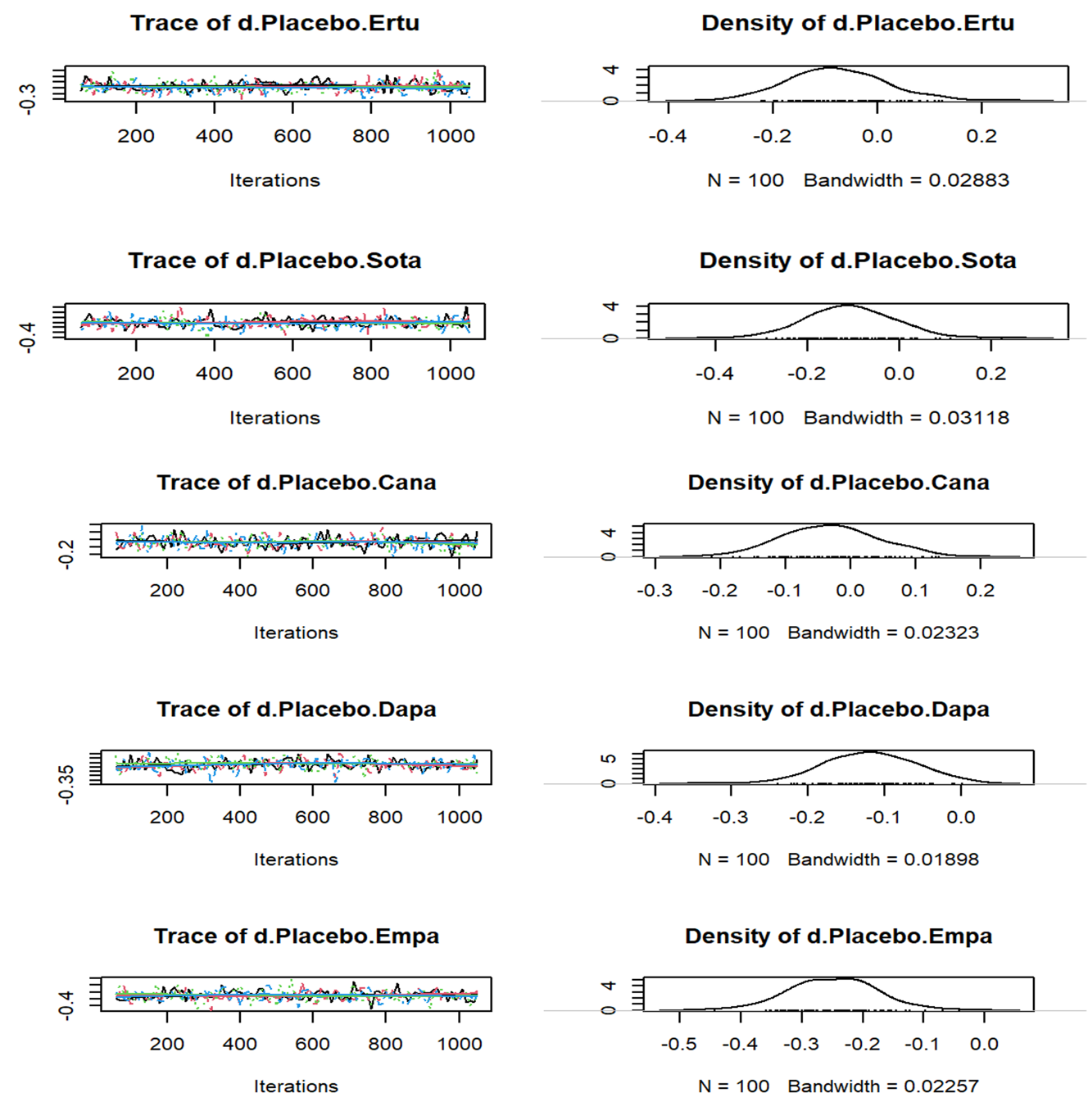


2. mcmc2 plot:


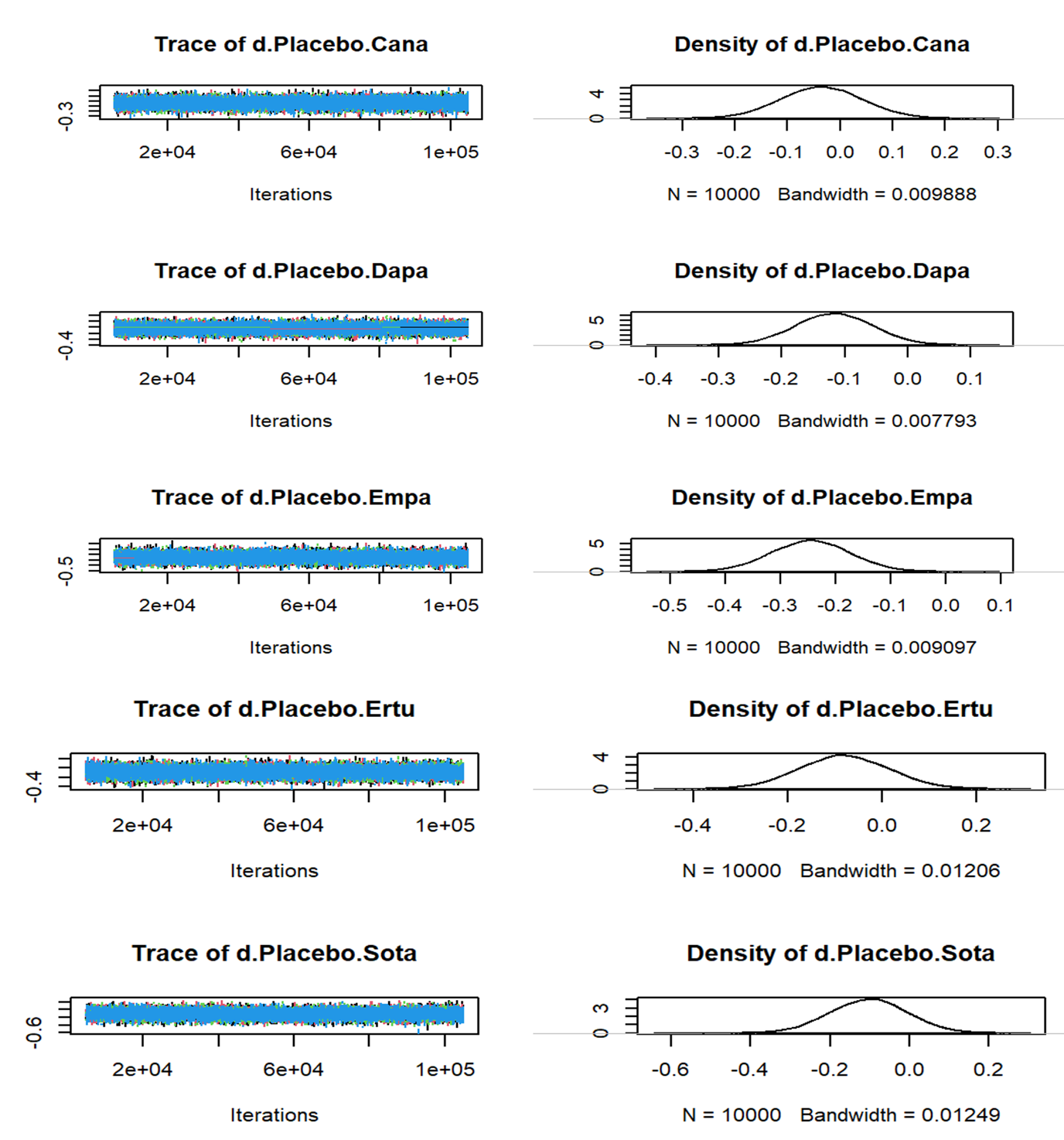


3. Gelman plot 1:


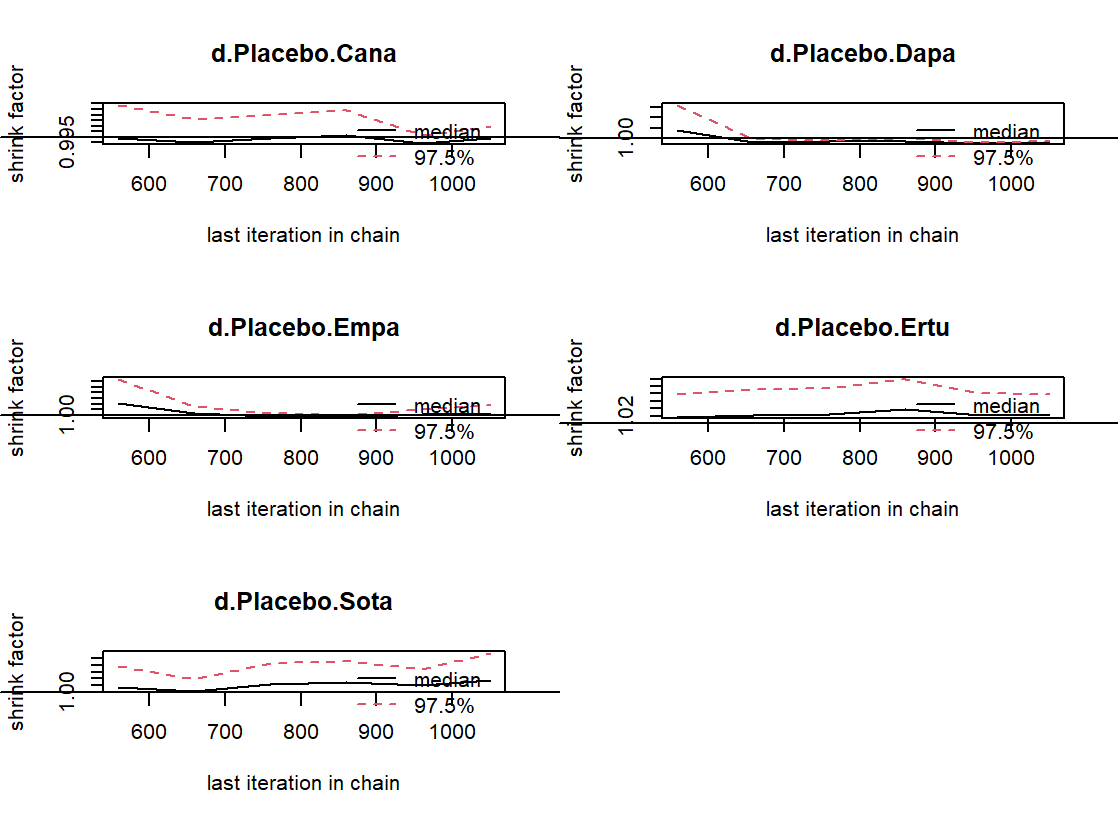


4. Gelman plot 2:


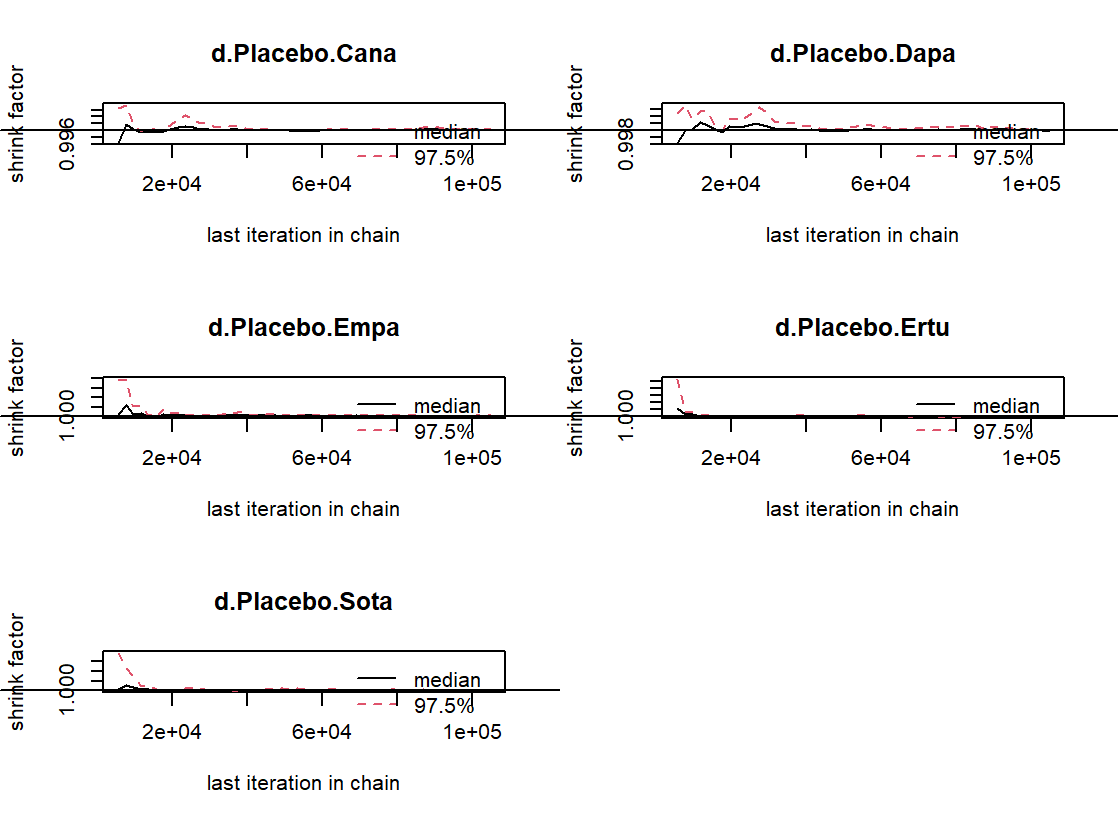


[E]. Leave one out sensitivity analysis:

| Study left out | Relative Risk (RR) | 95% Precision interval | Prediction interval |
| --- | --- | --- | --- |
| EMPA_KIDNEY | 0.91 | 0.82-1.01 | 0.65-1.28 |
| EMPAREG | 0.95 | 0.88-1.02 | 0.82-1.09 |
| EMPEROR_P | 0.89 | 0.80-1.00 | 0.63-1.28 |
| EMPEROR_R | 0.90 | 0.80-1.01 | 0.63-1.29 |
| DECLARE TIMI_58 | 0.89 | 0.80-1.00 | 0.62-1.28 |
| DAPA_HF | 0.88 | 0.80-0.97 | 0.66-1.17 |
| DAPA_CKD | 0.90 | 0.81-1.01 | 0.64-1.28 |
| DELIVER | 0.90 | 0.81-1.01 | 0.63-1.30 |
| CANVAS | 0.88 | 0.79-0.98 | 0.64-1.23 |
| CREDENCE | 0.91 | 0.82-1.02 | 0.65-1.29 |
| VERTIS_CV | 0.90 | 0.80-1.01 | 0.62-1.30 |
| SOLOIST_WHF | 0.90 | 0.81-1.01 | 0.63-1.28 |
| SCORED | 0.90 | 0.80-1.01 | 0.62-1.29 |
